# Dual actions of ANP on endothelial permeability

**DOI:** 10.1186/2050-6511-14-S1-O35

**Published:** 2013-08-29

**Authors:** Wen Chen, Heike Oberwinkler, Franziska Werner, Birgit Gaßner, Hitoshi Nakagawa, Robert Feil, Franz Hofmann, Jens Schlossmann, Alexander Dietrich, Thomas Gudermann, Motohiro Nishida, Sabrina Del Galdo, Thomas Wieland, Michaela Kuhn

**Affiliations:** 1 Institute of Physiology, University of Würzburg, Germany; 2 Interfakultäres Institut für Biochemie, University of Tübingen, Germany; 3 FOR 923, Technical University München, Germany; 4 Institute of Pharmacology and Toxicology, University of Regensburg, Germany; 5 Walther-Straub-Institute for Pharmacology and Toxicology, Ludwig-Maximilians-University München, Germany; 6 Department of Drug Discovery and Evolution, Graduate School of Pharmaceutical Sciences, Kyushu University, Higashi-ku, Fukuoka, Japan; 7 Institute of Experimental Pharmacology and Toxicology, Medical Faculty Mannheim, Heidelberg University; Germany

## 

Atrial natriuretic peptide (ANP), via its cGMP-forming guanylyl cyclase-A (GC-A) receptor, is critically involved in the regulation of arterial blood pressure and intravascular volume. To elucidate the role of the endothelial effects of ANP, we generated mice with conditional, endothelium (EC)-restricted ablation of the GC-A gene. Our observations in these and control mice demonstrated that ANP, via GC-A, mildly stimulates systemic transendothelial albumin transport in the microvasculature of skeletal muscle and skin. These and other studies indicate that concerted renal diuretic/natriuretic and mild endothelial hyperpermeability actions of ANP are essential to adjust intravascular fluid volume.

However, this notion apparently contradicts published *in vitro* and *in vivo* studies showing that ANP can strenghen the *pulmonary* barrier under inflammatory conditions, suggesting that the hormone either acts differently on pulmonary *vs* systemic endothelium, or it exerts opposite effects on quiescent endothelia (enhanced permeability) and an inflammation-activated endothelium (barrier stabilization). Endothelial hyperpermeability is characteristic of many *systemic* diseases, such as allergic responses, edema, and sepsis. In particular, histamine markedly and acutely enhances endothelial permeability. Activation of endothelial G_q_-coupled H_1_ receptors activates phospholipase C and elevates intracellular [Ca^2+^], ultimately leading to actin-myosin contraction and paracellular leakiness.

Here we combined studies in microvascular endothelial cells and intravital microscopy of vascular permeability in the m. cremaster microcirculation to study whether ANP counteracts not only pulmonary but also systemic inflammation, i.e. histamine-induced hyperpermeability. Our observations reveal that transient receptor potential canonical (TRPC) 6 channels mediate the inflammatory effects of histamine. Most importantly, they characterize a regulatory pathway by which histamine-induced activation of TRPC6 channels and subsequent calcium-dependent acute endothelial hyperpermeability are prevented by ANP/GC-A-induced, cGMP-dependent protein kinase I (cGKI) - mediated inhibitory phosphorylation of these channels.

